# Impact of Organelle Transport Deficits on Mitophagy and Autophagy in Niemann–Pick Disease Type C

**DOI:** 10.3390/cells11030507

**Published:** 2022-02-01

**Authors:** Maik Liedtke, Christin Völkner, Andreas Hermann, Moritz J. Frech

**Affiliations:** 1 Translational Neurodegeneration Section “Albrecht Kossel“, Department of Neurology, University Medical Center Rostock, 18147 Rostock, Germany; maik.liedtke@med.uni-rostock.de (M.L.); Christin.Voelkner@med.uni-rostock.de (C.V.); Andreas.Hermann@med.uni-rostock.de (A.H.); 2 Center for Transdisciplinary Neurosciences Rostock (CTNR), University Medical Center Rostock, 18147 Rostock, Germany; 3 Deutsches Zentrum für Neurodegenerative Erkrankungen (DZNE) Rostock/Greifswald, 18147 Rostock, Germany

**Keywords:** NPC1, NPC2, mitochondria, induced pluripotent stem cells, iPSCs, lysosomal storage disorder

## Abstract

Defective mitochondria are pathophysiological features of a number of neurodegenerative diseases. Here, we investigated mitochondrial dysfunction in the context of the rare lysosomal storage diseases Niemann–Pick disease type C1 and type C2 (NP-C1 and NP-C2). Mutations in either the *NPC1* or *NPC2* gene lead to cholesterol accumulation in late endosomes and lysosomes, resulting in impaired cholesterol homeostasis. The extent to which this may lead to mitochondrial dysfunction has been poorly studied so far. Therefore, we investigated the morphology, function, and transport of mitochondria, as well as their degradation via mitophagy, in a disease-associated human neural cell model of NP-C. By performing live cell imaging, we observed markedly reduced mitochondrial transport, although morphology and function were not appreciably altered. However, we observed a defective mitophagy induction shown by a reduced capability to elevate parkin expression and engulf mitochondria in autophagosomes after treatment with carbonyl cyanide 3-chlorophenylhydrazone (CCCP). This was accompanied by defects in autophagy induction, exhibited by a hampered p62 expression and progression, shown by increased LC3BII levels and a defective fusion of autophagosomes and lysosomes. The latter might have been additionally influenced by the observed reduced lysosomal transport. Hence, we hypothesized that a reduced recycling of mitochondria contributes to the pathophysiology of NP-C.

## 1. Introduction

Disturbances of mitochondrial morphology, movement, and function are pathophysiological features of a number of neurodegenerative diseases. In the present work, we investigated mitochondrial dysfunction in the context of the rare lysosomal storage diseases Niemann–Pick disease type C1 and type C2 (NP-C1 and NP-C2, respectively). NP-C is caused by mutations in the *NPC1* (95% of cases) or *NPC2* (5% of cases) gene, coding for the NPC1 and NPC2 proteins, which cooperate to transport cholesterol out of late endosomes and lysosomes, and therefore maintain the intracellular cholesterol homeostasis [1,2]. Mutations in one of the two genes result in a pathophysiological accumulation of cholesterol and sphingolipids in these organelles [3]. The heterogeneous clinical manifestations of NP-C1 and NP-C2 differ in regards of the onset of the disease, ranging from a severe early infantile form to a more mild form with disease onset in adulthood, and include neurological, psychiatric, and systemic symptoms [2]. On the cellular level, mitochondria are thought to have an impact on the progression of the disease. An increase in cholesterol in the mitochondrial membranes was described in a murine NP-C1 model, which was accompanied by a decreased membrane potential, reduced ATP synthase activity, and henceforth reduced ATP levels [4]. Comparable results regarding the cholesterol accumulation were acquired in patient-specific fibroblasts carrying mutations in the *NPC1* gene. Additionally, an altered organization of mitochondria, an increased respiration, and an altered composition of the respiratory chain complexes and decreased ATP levels were reported [5]. In addition to proper mitochondrial morphology and function, unimpeded transport of these organelles is essential for the maintenance of cellular metabolic homeostasis. A functional anterograde transport is necessary to maintain a proper energy supply and calcium buffering throughout the whole cell up to the distal parts of the axon and dendrites [6]. The retrograde transport, in turn, is an essential process for the degradation of damaged mitochondria via the proteasome or mitophagy to avoid the accumulation of damaged organelles and ensure the synthesis of new mitochondria [7]. Less is known about mitochondrial transport in general with respect to NP-C, whereas a contribution of reduced transport of organelles to the pathophysiology has been described for several other neurodegenerative diseases, such as amyotrophic lateral sclerosis (ALS) [8] or spinocerebellar ataxia 20 (SCAR20) [9].

A proper retrograde transport of mitochondria is substantial for their recycling. One of the main degradative pathways is macroautophagy, hereafter referred to as autophagy, in which different kinds of materials are degraded via their engulfment in membranous structures called autophagosomes. Autophagosomes fuse with lysosomes, and the enclosed material is degraded via lysosomal hydrolases to recycle macromolecules for biosynthetic pathways [10]. The degradation of mitochondria via mitophagy is initiated by the targeting of the damaged organelles by the serine/threonine kinase PTEN-induced kinase 1 (PINK1) together with the ubiquitin ligase parkin, leading to the incorporation into autophagosomes [11]. Defects of both autophagy and mitophagy have been described in NP-C fibroblasts and mice, as well as in various models of other neurodegenerative diseases, but the exact cause is not known. It has been suggested that a defective fusion of autophagosomes and lysosomes may be a reason, leading to an accumulation of autophagosomes and therefore the material being degraded, which in turn may cause cellular stress [12,13]. It was recently suggested that a reduced lysosomal transport, caused by the cholesterol accumulation in these organelles [14,15], resulted in the mislocalization of lysosomes, thus impacting the degradation process [16]. Therefore, we asked to what extent dysfunctional transport of organelles, such as mitochondria, autophagosomes, and lysosomes, may contribute to the pathophysiological cellular NP-C phenotype, especially with regard to autophagy and mitophagy. We investigated the mitochondrial morphology and function, as well as transport of mitochondria, autophagosomes, and lysosomes, by live cell imaging. The degradation of mitochondria by mitophagy, as well as general autophagy, was determined by immunofluorescence and colocalization analyses, as well as Western blot experiments using key proteins of both pathways. To achieve these goals, we used neural differentiated cells derived from NP-C1 and NP-C2 patient-specific induced pluripotent stem cells (iPSCs).

## 2. Materials and Methods

### 2.1. Neural Differentiation

Human fibroblast lines GM08398, GM18436, GM18453, and GM18455 were obtained from the NIGMS Human Genetic Cell Repository at the Coriell Institute for Medical Research, Camden, NJ, USA. GM08398 was used as control, and is hereafter referred to as ctrl. Cell line A113011, obtained from Centogene AG, Rostock, Germany, carries the homozygous *NPC1* mutation c.1180T>C, and is hereafter referred to as Mut A. Cell line GM18436 carries the compound heterozygous *NPC1* mutation c.1836A>C/c.1628delC, and is hereafter referred to as Mut B. Cell line GM18453 carries the homozygous *NPC1* mutation c.3182T>C, and is hereafter referred as Mut C. Cell line GM18455 carries the compound heterozygous *NPC2* mutation c.58G>T/c.140G>T, and is hereafter referred to as Mut D. Generation and characterization of iPSCs from patient-specific fibroblasts was published recently [17,18,19,20]. Neural differentiation of iPSCs was induced by density-dependent growing of iPSCs on Matrigel (Corning, NY, USA) to induce generation of neural rosettes. Once neural rosettes were formed spontaneously, neural progenitor cells (NPCs) were isolated using magnetic beads against the surface marker polysialylated-neural cell adhesion molecule (PSA-NCAM, Miltenyi Biotec, Bergisch Gladbach, Germany). NPCs were seeded at an expansion density of 100,000 cells/cm^2^ on poly-L-ornithine (PLO, 15 µg/mL; Sigma Aldrich, St. Louis, MO, USA)/laminin (10 µg/mL; Trevigen, Gaithersburg, MD, USA)-coated dishes in proliferation medium containing 40% Dulbecco’s Modified Eagle Medium (DMEM), 60% DMEM/F-12, 1X B27, 0.5% penicillin/streptomycin, 20 ng/mL basic fibroblast growth factor (FGF2, Amsbio, Abingdon, UK), and 20 ng/mL epidermal growth factor (EGF, Peprotech, Germany). For terminal neural differentiation into a mixed culture of ßIII-positive neurons and GFAP-positive astrocytes, cells were plated at a density of 45,000 cells/cm^2^ in differentiation medium containing 40% DMEM, 60% DMEM/F-12, 1X B27, and 0.5% penicillin/streptomycin, which was changed every 4 days over a period of 6 weeks.

### 2.2. Live Cell Staining and Imaging

For the staining of mitochondria, lysosomes, and autophagosomes in living cells, CellLight™ 2.0 BacMam technology from Thermo Fisher Scientific (Waltham, MA, USA) was used according to the manufacturer’s protocol. In brief, after 6 weeks of differentiation, an amount of CellLight™ Mitochondria-GFP, Lysosomes-RFP, Premo™ Autophagy Sensor LC3B-GFP, or Premo™ Autophagy Tandem Sensor RFP-GFP-LC3B Kit (all four from Thermo Fischer Scientific) equal to 35 particles per cell was diluted in culture medium, added to each well, and incubated for 24 h. Cells were then transformed to the imaging chambers containing EXS-HEPES buffer (151 mM NaCl, 1.25 mM NaH_2_PO_4_, 10 mM HEPES, 2.5 mM KCl, 2 mM CaCl_2_, 1 mM MgCl_2_, 10mM Glucose, pH 7.4), and live cell imaging was performed with a Zeiss AxioObserver microscope (Zeiss, Hamburg, Germany). Cells were kept at 37 °C during microscopy. Four to five videos with 300 frames and a frame rate of 4 frames per second were recorded of four independent experiments per cell line and condition.

### 2.3. Analysis of Organelle Transport

The NIH FIJI ImageJ software (National Institutes of Health, Bethesda, MD, USA) using the TrackMate v2.7.4 plugin was used to analyze the videos. Settings were as follows: pixel width: 0.23 μm; pixel height: 0.23 μm; voxel depth: 1 μm; crop settings: not applied; select a detector: DoG detector; estimated blob size: 1.6 μm; threshold: 2; median filter: no; subpixel localization: yes; initial thresholding: none; select view: HyperStack Displayer; set filters on spots: quality above 2; select a tracker: linear motion LAP tracker; initial search radius: 2 μm; search radius: 2 μm; max. frame gap: 2; set filters on tracks: track duration ≥ 3 s. All tracks were prefiltered to distinguish between moving and stationary organelles. A statistical analysis of the parameter “maximal distance” was performed, and the mean + SD of all tracked mitochondria in all cell lines was set as the threshold value. For mitochondria, every organelle that traveled further than 1 µm from its starting point was defined as “moving”. The same was done for lysosomes, leading to a threshold of 0.87 µm, and for autophagosomes (1.12 µm). The percentage of each population was calculated, and 10 different kinetic parameters of the moving fraction were analyzed. The parameters analyzed were:

- Track duration: time between the first and the last spot of a track;
- Mean speed: mean speed over the time course of track;
- Maximal speed: maximal speed between two spots of a track;
- Minimal speed: minimal speed between two spots of a track;
- Median speed: median of all velocities between two spots;
- Track displacement/track length: distance between the first and last spot of a track;
- Total distance travelled: sum of all distances between two consecutive spots;
- Maximal distance travelled: furthest distance from the starting point of a track;
- Confinement ratio:

(1) $$\mathrm{confinement}\;\mathrm{ratio}=\frac{\mathrm{track}\;\mathrm{length}}{\mathrm{total}\;\mathrm{distance}\;\mathrm{travelled}}$$

Formula (1): Confinement calculation. The confinement ratio was calculated using the TrackMate plugin in the FIJI ImageJ software.

Mean straight line speed: speed of the organelle moving at constant speed along a straight line from the first spot to the last spot of the track:

(2) $$\mathrm{mean}\;\mathrm{straight}\;\mathrm{line}\;\mathrm{speed}=\frac{\mathrm{track}\;\mathrm{length}}{\mathrm{track}\;\mathrm{duration}}$$

Formula (2): Calculation of the mean straight line speed. The mean straight line speed was calculated using the TrackMate plugin in the FIJI ImageJ software.

Additionally, the rate of stops per track was calculated. The analysis was conducted with R and GraphPad Prism 8.1 (GraphPad Software Inc., San Diego, CA, USA). The Z-score was calculated using Formula (3) [21]:

(3) $$Z(P_{X}{}^{\mathrm{Mut}})=\frac{\bar{P_{X}{}^{\mathrm{Mut}}}-\bar{P_{X}{}^{\mathrm{Ctrl}}}}{\frac{{\mathrm{SD}}_{X}^{\mathrm{Mut}}}{\sqrt{N_{X}^{\mathrm{Mut}}}}+\frac{{\mathrm{SD}}_{X}^{\mathrm{Ctrl}}}{\sqrt{N_{X}^{\mathrm{Ctrl}}}}}$$

Formula (3): Z-score calculation.

A Z-Score for each kinetic parameter was calculated according this formula to compare each NP-C deficient cell line with the control cell line. Abbreviations are as follows:

Z(P_X_^Mut^) = Z-Score of parameter x (e.g., mean speed) of the NPC1-deficient cell line (Mut) compared to the control cell line (ctrl). P_X_^Mut^ = mean of parameter X of the NPC1-deficient cell line;
P_X_^Ctrl^ = mean of parameter X of the control cell line;
SD_X_^Mut^ = standard deviation of parameter X of the NPC1-deficient cell line;
SD_X_^Ctrl^ = standard deviation of parameter X of the control cell line;
N_X_^Mut^ = sample size of parameter X of the NPC1-deficient cell line;
N_X_^Ctrl^ = sample size of parameter X of the control cell line.

### 2.4. Immunofluorescence and Fluorescence Microscopy

For immunofluorescence microscopy, cells were grown on coverslips coated with PLO/laminin. Cells were washed with phosphate-buffered saline (PBS) with calcium and magnesium (PBS+/+) and fixed with 4% paraformaldehyde (PFA) for 10 min on ice. Subsequently, cells were washed with PBS+/+ and incubated with permeabilization buffer containing 0.1% Triton X-100 in PBS for 10 min on ice. After a washing step with PBS+/+, cells were incubated with 1% normal goat serum (NGS, Santa Clara, CA, USA) for 1 h at room temperature, followed by incubation with primary antibodies in 3% NGS at 4 °C overnight. The antibody used was mouse anti-Tom20 (1:100, Santa Cruz Biotechnology). Next, cells were washed with PBS+/+, followed by incubation with antimouse IgG conjugated with the Alexa488 secondary antibody (Invitrogen, Carlsbad, CA, USA) diluted 1:100 for 2 h at room temperature. After washing with PBS+/+, nuclei were stained with DAPI (5 min, 250 ng/mL). Cover slips were mounted using Fluoromount-G^®^ (SouthernBiotech, Birmingham, AL, USA). Microscopy was performed using an LSM 900 laser scanning microscope (LSM) equipped with a 130 × 100 STEP motorized scanning stage; a URGB laser module with a 405 nm, 488 nm, and 561 nm diode laser; a Plan-APOCHROMAT 63×/1.4 oil objective, and a GaAsP-PMT detector, using the ZEN 2 blue edition imaging software (all Zeiss, Hamburg, Germany). 

### 2.5. Colocalization Analysis

For colocalization analysis, cells grown on coverslips were transduced with either the CellLight™ BacMam 2.0 technology for mitochondria and autophagosomes or the Premo™ Autophagy Tandem Sensor RFP-GFP-LC3B Kit (all Thermo Fisher Scientific, Waltham, MA, USA), and fixed with 4% PFA afterwards. From each of 4 independent experiments, 10 pictures were taken with the LSM 900, and a colocalization analysis was performed using NIH ImageJ software and the Just another Colocalization Plugin (JaCoP) to determine the Pearson’s correlation coefficient.

### 2.6. Determination of Mitochondrial Morphology

For the analysis of mitochondrial morphology, cells were stained with mouse anti-Tom20 antibody as described in Section 2.4. For each cell line, 30 pictures of 3 independent experiments were taken, and the morphology was analyzed with the Shape Descriptor plugin in the FIJI ImageJ software. The form factor and aspect ratio were calculated according to Formulas (4) and (5):

(4) $$\mathrm{form}\;\mathrm{factor}=\frac{p^{2}}{4\times \pi \times A^{2}}$$

Formula (4): Calculation of the form factor of mitochondria. The mitochondrial form factor was calculated as the ratio between the perimeter (p) and the area (A). Parameters were acquired using the Shape Descriptor plugin in the FIJI ImageJ software.

(5) $$\mathrm{aspect}\;\mathrm{ratio}=\frac{M}{m}$$

Formula (5): Calculation of the aspect ratio of mitochondria. The mitochondrial aspect ratio was calculated as the ratio between the longitudinal (M) and width axis (m). Parameters were acquired using the Shape Descriptor plugin in the FIJI ImageJ software.

### 2.7. Western Blot Analysis

Western blot was performed as recently described [22]. In brief, whole cell lysates were prepared by incubation of the cells for 30 min on ice in RIPA-lysis buffer (in mM: TRIS 20, NaCl 137, sodium deoxycholate 12, EDTA 2, supplemented with 0.1% SDS, 1% Triton^®^ X-100, 10% glycerol with cOmplete™, Mini, EDTA-free Protease Inhibitor Cocktail and PhosStop™ phosphatase inhibitor (both Roche Diagnostics GmbH, Mannheim, Germany)). Lysates were centrifuged at 15,000× *g* for 25 min at 4 °C. Protein concentration of the supernatant was determined using the Pierce™ BCA Protein Assay Kit (Thermo Fisher Scientific, Waltham, MA, USA) according to the manufacturer´s instructions. Samples were boiled for 5 min at 95 °C in 5× Laemmli-buffer (125 mM TRIS, 20% glycerol, 2% SDS, 5% β-mercaptoethanol, 10% bromphenol blue) and subsequently centrifuged at 22,000× *g* for 1 min at 4 °C. For electrophoresis, Criterion™ Vertical Electrophoresis Cell with Criterion™ TGX Stain-Free™ Precast Gels (4–15%) (Bio-Rad Laboratories, Hercules, CA, USA) were used. The electrophoresis buffer contained 250 mM TRIS, 2 M glycine, and 0.1% SDS. For the Western blot, the Trans-Blot^®^ Turbo™ Transfer System with Trans-Bolt^®^ Turbo™ Transfer Pack (Midi Format, 0.2µm nitrocellulose, Bio-Rad Laboratories, Hercules, CA, USA) was used. Afterward, the membranes were washed in TRIS-buffered saline (TBS, 20 mM TRIS and 137 mM NaCl (pH 7.5)) for 5 min and blocked with 5% bovine serum albumin (BSA, Carl Roth GmbH & Co. KG, Karlsruhe, Germany) in TBS supplemented with 0.1% Tween^®^ 20 (TBST) for 1 h. For protein detection of Tom20 (mouse, 1:1000, Santa Cruz Biotechnology), Hsp60 (rabbit, 1:1000, Abcam, Cambridge, UK), parkin (mouse, 1:500, Santa Cruz Biotechnology, Dallas, TX, USA), LC3BII/I (rabbit, 1:1000, Cell Signaling Technology, Cambridge, UK), and SQSTM1/p62 (Sigma Aldrich, St. Louis, MO, USA), membranes were incubated with primary antibody solution (3% BSA in TBST) overnight at 4 °C and for 1 h at room temperature for detection of GAPDH (mouse, 1:10.000, Abcam, Cambridge, UK). Subsequently, membranes were washed 3× with TBST and incubated for 1 h with DyLight™ secondary antibodies (Thermo Fisher Scientific, Waltham, MA, USA). Precision Plus Protein Dual Xtra Standards (Bio-Rad Laboratories, Hercules, CA, USA) was used as a molecular weight marker. Finally, membranes were washed 3× with TBST and 1× with TBS and dried. The Odyssey Infrared Imaging System (LI-COR Biosciences GmbH, Bad Homburg, Germany) was used to visualize and quantify the protein signals. Expression of GAPDH was used to normalize the expression of Tom20, Hsp60, p62, and parkin. The expression of LC3BII was normalized on its cytosolic form, LC3BI. 

### 2.8. Analysis of Mitochondrial Membrane Potential

For measuring the mitochondrial membrane potential, the JC-10 from AAT Bioquest (Sunnyvale, CA, USA) was used. Cells were cultivated in black 96-well plates for 6 weeks. Cells were washed with Hank’s balanced salt solution (HBSS) and incubated with 20 µM JC-10 in HBSS (Thermo Fisher Scientific, Waltham, MA, USA)/EXS-HEPES (1:1) for 45 min, and the fluorescence intensity at Ex/Em = 490/525 nm and 540/590 nm was measured with a Spark^®^ multimode microplate reader (TECAN, Hamburg, Germany). Graphs show the calculated ratio of Em 590/525.

### 2.9. Statistical Analysis

Data are presented as mean ± SD of at least three independent experiments. Statistical analysis was performed with GraphPad Prism 8.1 (GraphPad Software Inc., San Diego, CA, USA). The D’Agostino–Pearson normality test was used to test the data sets for normal distribution. When applicable, the statistical significance was tested by using an unpaired t-test or ordinary one-way ANOVA test with Dunnett’s multiple comparisons test. Otherwise, a Two-way ANOVA with Sidak’s multiple comparisons test, a nonparametric Mann–Whitney test or Kruskal–Wallis rank tests was used. The *p*-values were considered statistically significant when * = *p* < 0.05, * = *p* < 0.01, *** = *p* < 0.001 when tested against the untreated control, or # = *p* < 0.05, ## = *p* < 0.01 when tested against the treated control.

## 3. Results

### 3.1. Mitochondria Were Smaller, but Showed No Differences in Function

**Figure 1:**
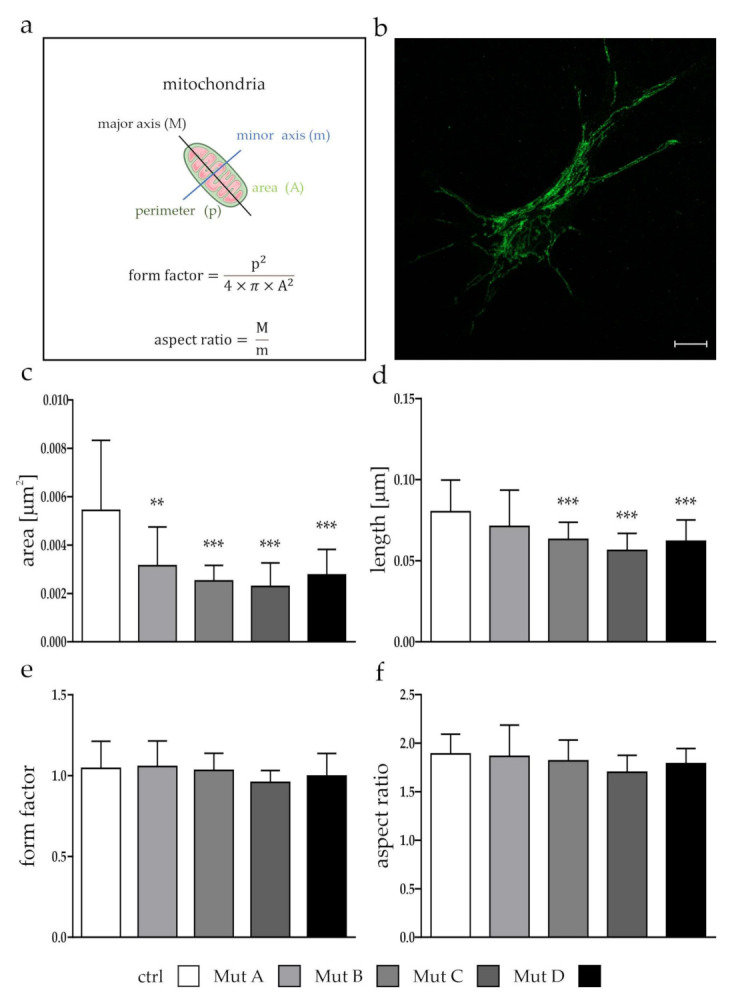
Analysis of mitochondrial morphology. Length (major axis), area, form factor, and aspect ratio of 30 mitochondria from 3 independent experiments were analyzed using immunofluorescence stainings of Tom20. (**a**) Schematic description of the used parameters. Graphic was created with Microsoft PowerPoint. For the graphical representation of the mitochondrion, a template from Smart (Servier Medical Art) [23] was used. (**b**) Example of a Tom20 staining that was used to evaluate mitochondrial size and shape. Scalebar = 10 µm. (**c**) Mitochondrial area was reduced in all NPC-deficient cell lines. (**d**) The length of mitochondria were reduced, whereas one cell line failed to meet statistical significance. No differences could be observed regarding the calculated (**e**) form factor and (**f**) aspect ratio of mitochondria. Graphs show mean ± SD with *N* = 3, *n* = 30. One-way ANOVA and a Kruskal–Wallis post hoc test were used to determine statistical significance, with ** = *p* < 0.01, *** = *p* < 0.001. ctrl = control cell line. Mut A (NP-C1): c.1180T>C; Mut B (NP-C1): c.1836A>C/c.1628delC; Mut C (NP-C1): c.3182T>C; Mut D (NP-C2): c.58G>T/c.140G>T.

The first step of our evaluation of mitochondria in NP-C was to look for their gross morphology. By using the Shape Descriptor plugin in ImageJ, we determined the area and length, and calculated the form factor and the aspect ratio (Figure 1a) of mitochondria stained with an antimitochondrial import receptor subunit TOM20 homolog (Tom20) antibody (Figure 1b; Supplementary Figure S1) from 30 randomly selected cells of three independent experiments for each cell line.

**Figure 2:**
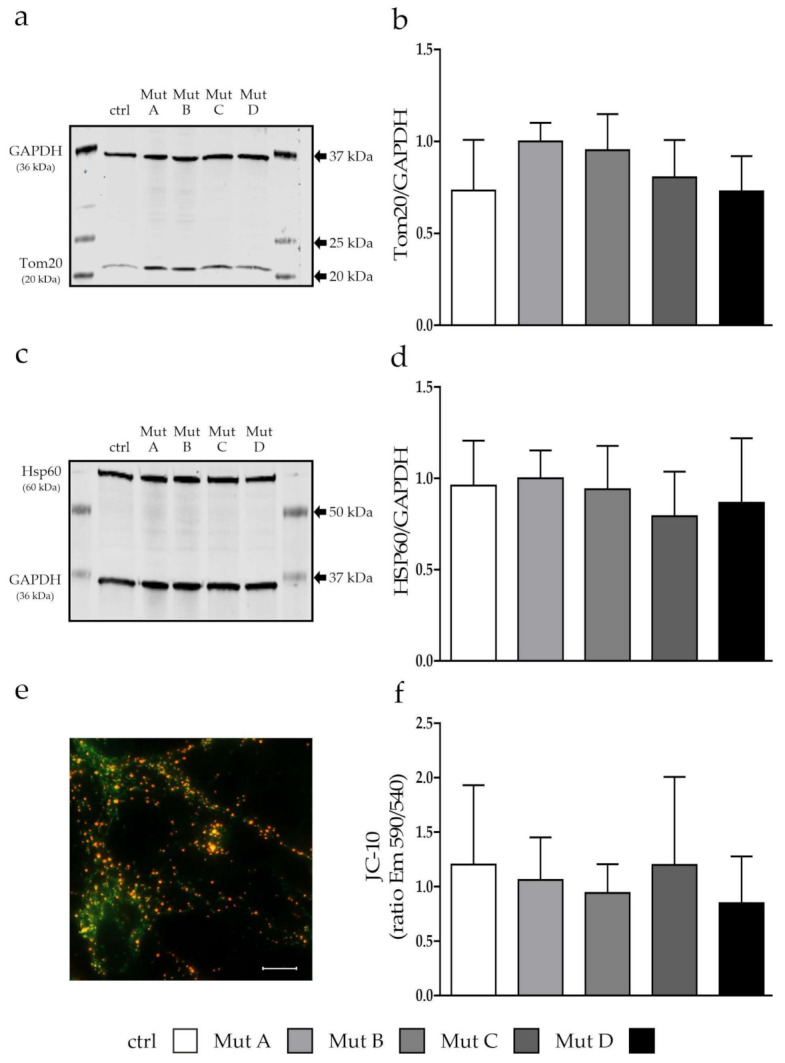
Evaluation of mitochondrial health. (**a**) Example of a Western blot for the mitochondrial marker Tom20 (20 kDa). (**b)** Quantification of the Tom20 protein expression showed no differences, whereas two NPC1-deficient cell lines showed a slight increase. (**c**) Example of Western blot of the mitochondrial heat-shock protein Hsp60 (60 kDa). (**d**) Quantification of Hsp60 revealed no differences between the NPC-deficient and the control cell lines. (**e**) Example of living cells stained with the MMP marker JC-10. Scalebar = 10 µm. (**f**) MMP was not affected in NPC-deficient cells. Graphs show mean ± SD with *N* = 4, *n* = 6–11 for (**b**) and (**d**), and *N* = 5 and *n* = 20 for (**f**). ctrl = control cell line. Mut A (NP-C1): c.1180T>C; Mut B (NP-C1): c.1836A>C/c.1628delC; Mut C (NP-C1): c.3182T>C; Mut D (NP-C2): c.58G>T/c.140G>T.

Mitochondria showed an overall reduced size (area, Figure 1c), length (major axis, Figure 1d), and width (minor axis, data not shown), whereas no differences could be observed between the NPC1- and NPC2-deficient cell lines and the control cell line using the form factor (Figure 1e) and aspect ratio (Figure 1f). As a reduced size might have had an impact on the functionality, we evaluated the number of organelles (Figure 2a,b), their stress levels (Figure 2c,d), and their membrane potentials (Figure 2e,f). We used Tom20, a protein located in the mitochondrial outer membrane, to evaluate the amount of mitochondria in the cells, and found it slightly but not significantly increased in two NPC1-deficient cell lines (Mut A and Mut B; Figure 2a,b). To analyze mitochondrial stress, we determined the level of the mitochondria-specific heat-shock protein Hsp60, which is necessary for the proper folding of proteins inside the mitochondria [24]. We found no differences in the expression level of Hsp60, indicating that the mitochondria in neither NPC1- nor NPC2-deficient cells suffered from stress (Figure 2c,d). JC-10 was used to stain mitochondria with regard to their membrane potential (MMP). Once more, no differences could be observed between the NPC1- and NPC2-deficient cell lines and the control cell line (Figure 2e,f; Supplementary Figure S2), which led us to conclude that the overall mitochondrial function seemed not to be affected, although the mitochondria seemed to be significantly smaller in the NPC-deficient cells. 

### 3.2. The Transport Speed of Moving Mitochondria was Reduced Due to an Increased Amount of Stops

**Figure 3:**
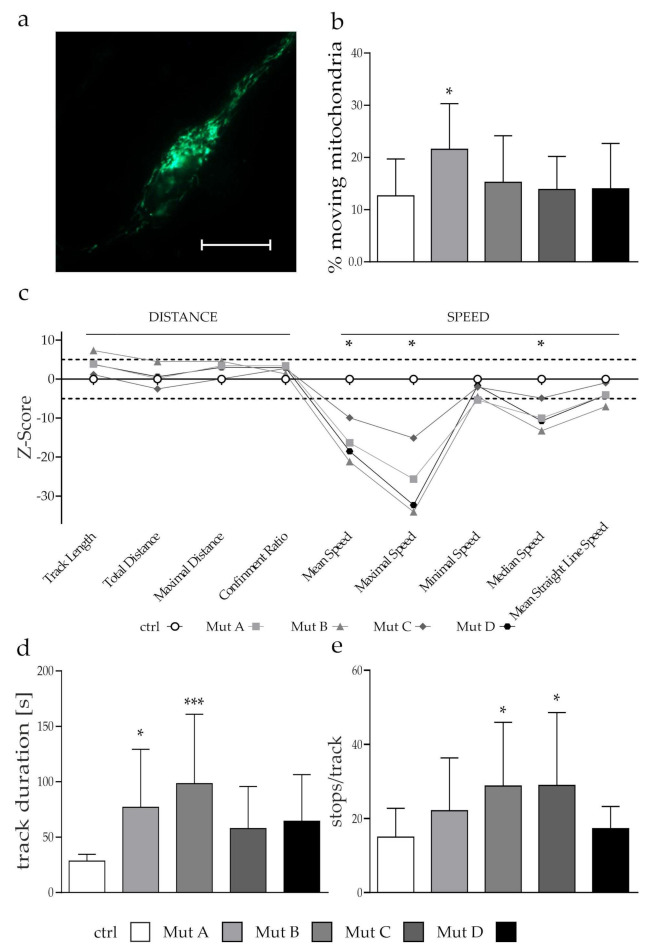
Analysis of mitochondrial transport. (**a**) Example of neural cells transduced with the CellLight^TM^ BacMam 2.0 technology for mitochondria. Scalebar = 20 µm. (**b**) Analysis revealed that 10–30% of all mitochondria were moving. Mut A showed a significantly increased percentage of moving mitochondria compared to the control cell line. (**c**) Z-score for each of the speed and distance parameters was calculated using the formula in Section 2.3. A Z-score higher than 5 and lower than –5 was defined as significantly different from the control cell line (dashed lines). The symbols of the individual parameters are connected with a line. This was not intended to indicate any relationship between the individual parameters, but only serves to improve the interpretability of the data presented. The traveled distance seemed not to be affected, whereas three speed parameters were reduced. (**d**) Bar graph of the track duration showing a significant increase in Mut A and B. (**e**) Bar graph of the calculated amount of stops/track. All three NPC1-deficient cell lines showed an increased number of stops, but only two were statistically significant. Graphs show mean ± SD with *N* = 3, *n* = 30. One-way ANOVA and a Kruskal–Wallis post hoc test were used to determine statistical significance, with * = *p* < 0.05, *** = *p* < 0.001. ctrl = control cell line. Mut A (NP-C1): c.1180T>C; Mut B (NP-C1): c.1836A>C/c.1628delC; Mut C (NP-C1): c.3182T>C; Mut D (NP-C2): c.58G>T/c.140G>T.

Proper mitochondrial transport is a key element for a functional cellular metabolism, whereas dysfunctional transport has been described for a number of neurodegenerative diseases [25]. The mitochondrial network needs to be tightly regulated to ensure a proper energy supply, calcium buffering, and defense against reactive oxygen species (ROS) throughout the cell. Therefore, mitochondrial transport and localization is a key mechanism, especially for postmitotic and arborized cells such as neurons. However, little is known about organelle transport in NP-C. Therefore, we investigated the kinetics of mitochondrial transport using live cell imaging (Figure 3a). First, we used the “maximal distance” as a parameter to set a cut-off value between moving and stationary mitochondria. A statistical approach (see Section 2.3) was used, and a maximal distance from the starting point of more than 1 µm during the movement was defined as a moving organelle. The determination of the percentage of moving mitochondria revealed that 10–30% of mitochondria were moving, which was in accordance with findings in neurons of different other model organisms [26]. One of the NPC1-deficient cell lines showed a significant increase in the percentage of moving mitochondria (Figure 3b). Further analyses of the kinetics were performed with the moving fraction of mitochondria to obtain data on their speed (mean speed, minimal speed, maximal speed, median speed, mean straight line speed), distance (track length, total distance traveled, maximal distance traveled, confinement ratio) and time (track duration). To obtain an overview of the movement pattern of the mitochondria, we used 9 of the 10 mentioned parameters (except the track duration) and calculated a Z-score (Figure 3c) for each parameter (formula shown in Section 2.3). Calculations were done as previously reported by Pal et al., 2018 [21].

An evaluation of the transport kinetics revealed that three of the speed parameters (mean speed, maximal speed, and median speed) were reduced in comparison to the control cell line; whereas the distance parameters, such as track length, total and maximal distance, and confinement ratio, were unaffected (Figure 3c). This led to the increased track duration (Figure 3d) and allowed us to hypothesize that the mitochondria reached their predetermined spot, but needed a longer time span for that due to an overall reduced speed. As the mitochondrial movement was not linear and was characterized by direction changes and stops, we calculated the amount of stops and the direction changes of each organelle during their movement. A velocity (calculated by TrackMate) below 0.05 µm/s between two time points was defined as a “stop”, and the amount of stops per track was counted. We found an increased amount of stops in all three NPC1-deficient cell lines, whereas only two of them were significantly different (Figure 3e). Additionally, the amount of direction changes was reduced (data not shown), which could be an explanation for the increased time span and the reduced overall speed.

In case of mitochondrial damage, their degradation and recycling is a crucial step to maintain a proper energy supply in the cell, as well as to prevent the cell from apoptosis. As a reduced transport efficiency may influence the recycling process of mitochondria, the next step was to investigate whether the transport deficiencies had an impact on one of the degradation pathways of the mitochondria, namely mitophagy.

### 3.3. Degradation of Mitochondria via Mitophagy Is Reduced

A specific form of autophagy is the so-called mitophagy, displaying a degradation pathway for mitochondria in which damaged organelles are engulfed in autophagosomes and transported to lysosomes, where the degradation occurs. We analyzed the degradation of mitochondria via this pathway under physiological conditions, as well as after the induction of mitochondrial damage with carbonyl cyanide chlorophenylhydrazone (CCCP), a mitochondrial uncoupling agent. CCCP reduces the mitochondrial membrane potential by increasing the proton permeability of the mitochondrial inner membrane, leading to their degradation via mitophagy [27]. 

**Figure 4:**
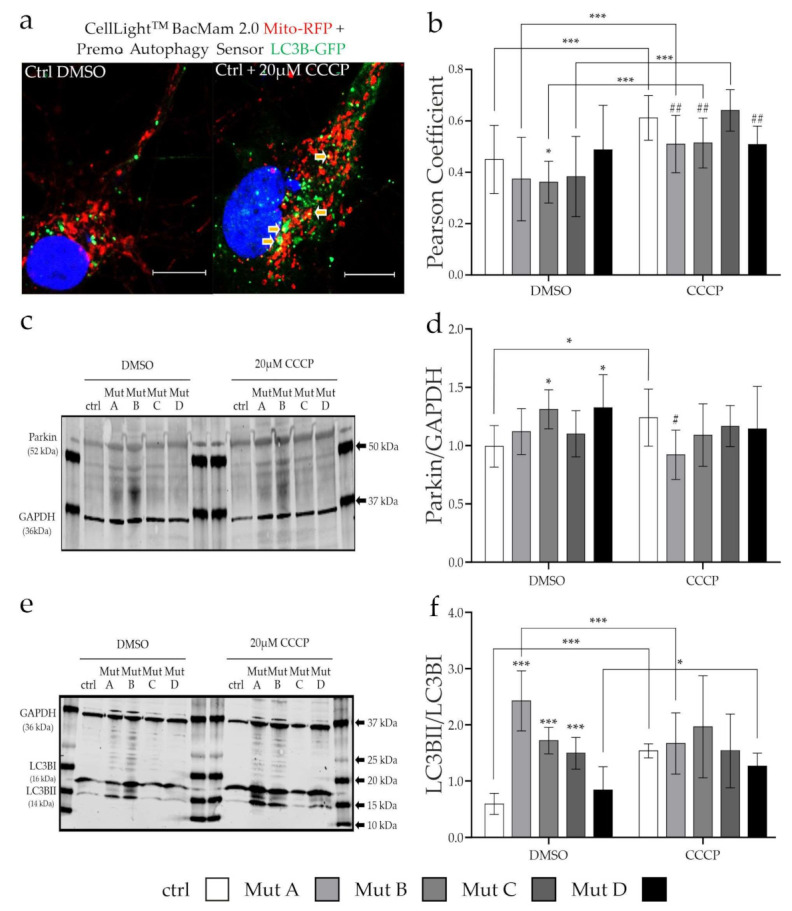
Analysis of the induction of mitophagy with CCCP. (**a**) Example cells transduced with CellLight^TM^ BacMam 2.0 MitoRFP and LC3B-GFP of the control cell line, without treatment (DMSO) and with treatment with carbonyl cyanide chlorophenylhydrazone (CCCP). Arrows indicate autophagosomes with enclosed mitochondria. Scalebar = 20 µm. (**b**) Analysis of colocalization of mitochondria and autophagosomes was performed on the pictures shown in (**a**). The three NPC1-deficient cell lines tended to show a reduced colocalization, but only one was significantly reduced. After CCCP treatment, the reduction was even more pronounced. *N* = 3, *n* = 30. (**c**) Parkin (52 kDa) expression was determined by Western blot. (**d**) An increased parkin level could be detected in Mut B and Mut D. After CCCP treatment, almost all NP-C cell lines showed a reduced parkin level compared to the control cell line. *N* = 4, *n* = 7–11. (**e**) Example Western blot of LC3B with the two isoforms LC3BI (16 kDa) and LC3BII (14kDa). (**f**) Quantification of Western blot shown in (**e**). All three NPC1-deficient cell lines showed an increased LC3BII/LC3BI ratio. After CCCP treatment, no differences between the cell lines could be observed. *N* = 4, *n* = 7–12. Graphs show mean ± SD. Two-way ANOVA with Sidak’s multiple comparisons test was used to determine statistical significance. Statistical significance against the untreated control is indicated by (*), with * = *p* < 0.05, *** = *p* < 0.001; and against the treated control by (#), with # = *p* < 0.05, ## = *p* < 0.01. ctrl = control cell line. Mut A (NP-C1): c.1180T>C; Mut B (NP-C1): c.1836A>C/c.1628delC; Mut C (NP-C1): c.3182T>C; Mut D (NP-C2): c.58G>T/c.140G>T.

First of all, we determined the general capability of the cells to induce mitophagy by evaluation of the engulfment of mitochondria into autophagosomes. Our analysis showed a slight reduction in the colocalization of mitochondria and autophagosomes in the three NPC1-deficient cell lines under basal conditions (DMSO; Figure 4a,b; Supplementary Figure S3), although only one of them was significantly different from the control cell line. Treatment with CCCP for 6 h increased the colocalization of mitochondria and autophagosomes in all but one (Mut D) cell line, indicating an induction of mitophagy. Interestingly, the increase in Mut A, Mut B, and Mut D was not as efficient as in the control cell line (Figure 4b, CCCP), pointing at a reduced capability to induce mitophagy in these cell lines.

As mitophagy is initiated by parkin, an E3 ubiquitin ligase that is recruited to mitochondria and binds to the mitochondrial rho GTPase 1 and/or 2 (Miro1 and 2) for ubiquitination and targets them for degradation [11,28], we evaluated the expression of this ligase with and without mitochondrial damage. Under basal conditions (DMSO), the expression level of Parkin was significantly increased in Mut B and Mut D, which may have indicated an increased need of these cells to degrade mitochondria. Only the control cell line was able to respond to the induction of mitochondrial damage by CCCP. This was reflected by an increase in parkin expression, where parkin is described to induce mitophagy (Figure 4d). This might explain the reduced capability of the NPC-deficient cells to degrade mitochondria, as their targeting by parkin, especially after induction of mitochondrial damage, seemed to be less efficient than in the control cell line. As this process required the formation of new autophagosomes, the next step was to evaluate this part of the process.

To this end, we quantified the expression of the autophagosomal marker microtubule-associated proteins 1A/1B light chain 3B (LC3B). During autophagy induction, the cytosolic LC3BI is converted to LC3BII, which is incorporated into the autophagosomal membrane. Therefore, the LC3BII/LC3BI ratio is a suitable marker for the evaluation of autophagosome formation. Under basal conditions, the three NPC1-deficient cell lines showed an increased LC3BII/LC3BI ratio (Figure 4e,f), which was in accordance with previous findings in NPC1-deficient cells and other neurodegenerative diseases, pointing at a defective clearance of autophagic vesicles [29]. After treatment with CCCP, no differences in the LC3BII/LC3BI ratio could be observed in the mutant lines, while the control line showed a higher LC3BII/I ratio compared to the baseline condition, indicating that only the control cell line was able to respond to the mitochondrial damage with the induction of autophagosome formation. In contrast, the NPC1-deficient cell lines may have already reached the maximal amount of autophagosomes, possibly due to deficient clearance of these vesicles. Because the results suggested a general defect in autophagosome recycling, and thus a reduced ability to induce mitophagy, we were next interested in analyzing autophagy.

### 3.4. Initiation and Progression of Autophagy Was Affected 

**Figure 5:**
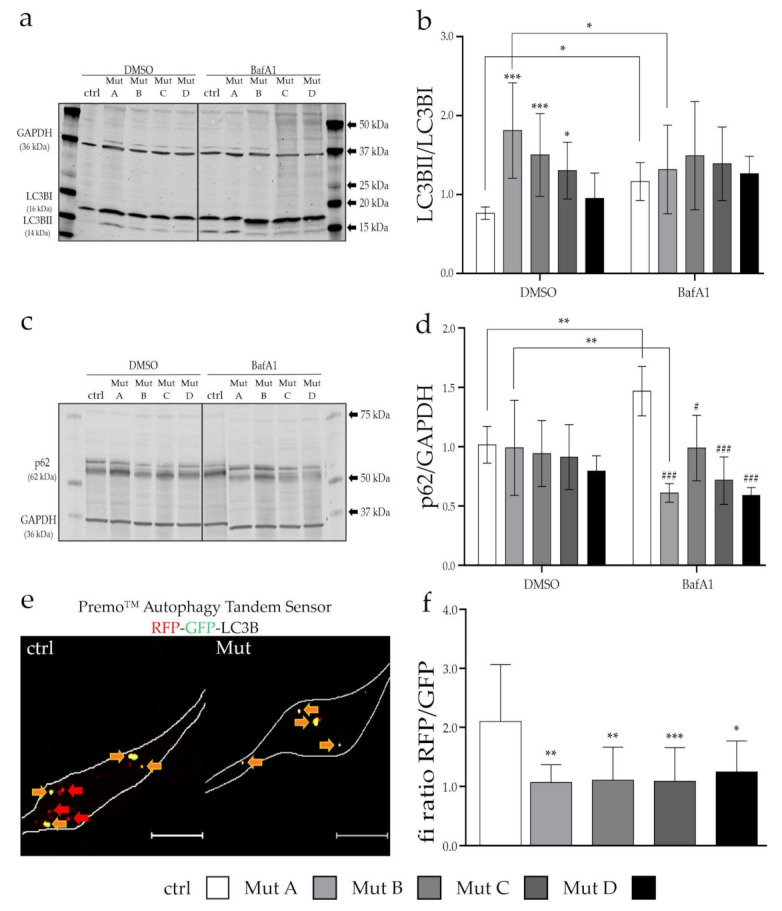
Analysis of the initiation and progression of the autophagic pathway. (**a**) Example Western blot for LC3B with the two isoforms LC3BI (16 kDa) and LC3BII (14kDa), without treatment (DMSO) and with treatment with bafilomycin A1 (BafA1). (**b**) Quantification of LC3BI and II with and without BafA1. Only the control cells showed an increase in the LC3BII/LC3BI ratio when blocking the clearance with BafA1. *N* = 4, *n* = 6–8. (**c**) Example Western blot of p62 (62 kDa) with and without BafA1 treatment. (**d**) Quantification of p62 showed that again only the control cell line was able to increase the p62 expression after BafA1 treatment. *N* = 4, *n* = 6–9. (**e**) Example picture of the control cell line transduced with the Premo™ Autophagy Tandem Sensor RFP-GFP-LC3B. Scalebar = 20 µm. (**f**) Quantification of colocalization, based on the fluorescence intensity (fi) ratio of RFP/GFP, showed a reduced ratio in all NPC-deficient cells. Graphs show mean ± SD with * = *p* < 0.05, ** = *p* < 0.01, *** = *p* < 0.001 vs. untreated ctrl; and # = *p* < 0.05, ### = *p* < 0.001 vs. untreated of the same cell line. ctrl = control cell line. For (**b**) and (**d**), two-way ANOVA with Sidak’s multiple comparisons test was used; and for (**f**), one-way ANOVA with Dunnett’s multiple comparisons test was used to determine statistical significance. Statistical significance against the untreated control is indicated by (*), with * = *p* < 0.05, ** = *p* < 0.01, *** = *p* < 0.001; and against the treated control by (#), with # = *p* < 0.05, ### = *p* < 0.01. Mut A (NP-C1): c.1180T>C; Mut B (NP-C1): c.1836A>C/c.1628delC; Mut C (NP-C1): c.3182T>C; Mut D (NP-C2): c.58G>T/c.140G>T.

As mitophagy is a specialized form of general autophagy, and it is known that autophagy is somehow affected in NP-C, we added a more general analysis of autophagy to our study. We analyzed the autophagosome formation, the targeting of the material to be degraded, and the fusion of autophagosomes with lysosomes, as these were the crucial steps for the degradation of the cargo (Figure 5). 

To evaluate the formation of autophagosomes, we determined the LC3BII/LC3BI ratio after blocking their degradation for 6 h with bafilomycin A1 (bafA1). BafA1 inhibits the vacuolar H^+^-ATPase (V-ATPase), thereby depriving acidic compartments such as lysosomes of H^+^ ions, leading to an increase in their pH. This leads to a functional inhibition of lysosomal hydrolases and lysosomes themselves, and will cause an accumulation of autophagosomes. As shown in Figure 5a,b, despite having an increased basal LC3BII/LC3BI ratio, none of the NPC1- or NPC2-deficient cell lines were able to further increase the LC3BII/LC3BI ratio after bafA1 treatment. This allowed us to assume that not only had the autophagosome synthesis already reached their maximum capacity, but also that the clearance of autophagic vesicles may have been impaired.

Another marker for autophagy is sequestosome-1 (SQSTM1), also known as the ubiquitin-binding protein p62. During autophagy induction, p62 binds to the material to be degraded as well as LC3BII to initiate its engulfment into the forming autophagosomes, and targets the cargo for degradation. Under basal conditions, no differences could be observed between NPC-deficient cells and the control cell line (Figure 5c,d). The bafA1 treatment led to an increase in the p62 level in the control cell line, whereas the NPC1- and NPC2-deficient cells did not show any differences, supporting the hypothesis of a defect in the cargo-targeting step.

A crucial step during autophagy is the fusion of autophagosomes and lysosomes to form so-called autolysosomes. To evaluate this process, we transduced the cells with a tandem sensor of LC3B labeled with GFP and RFP. As autophagosomes appeared yellow, the pH reduction after fusion of the autophagosomes with lysosomes quenched the GFP signal, leading to red fluorescent autolysosomes. In that case, the ratio of the fluorescence intensity of the RFP and GFP signal allowed us to evaluate the amount of formed autolysosomes, and hence the fusion of the two organelles (Figure 5e,f; Supplementary Figure S4). We found a significantly reduced RFP/GFP ratio in all NPC-deficient cell lines, which was accompanied by a decreased amount of autolysosomes (Figure 5f), indicating a disturbed fusion process. This also highlighted the additional defect in the later steps of the degradation pathway impairing the recycling of cargo, especially mitochondria.

### 3.5. Lysosomes but Not Autophagosomes Showed a Tendency of a Reduced Transport

**Figure 6:**
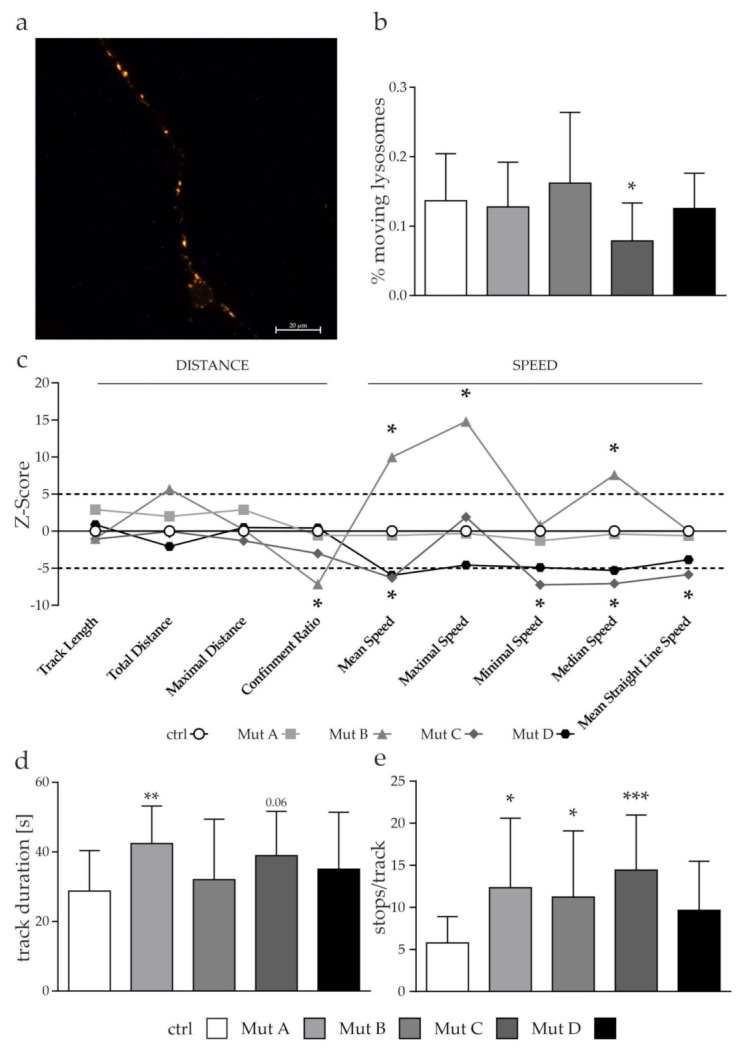
Analysis of lysosomal transport. (**a**) Example picture of cells transduced with the CellLight^TM^ BacMam 2.0 technology for lysosomes. Scalebar = 20 µm. (**b**) The percentage of moving lysosomes was reduced in one NPC1-deficient cell line. (**c**) Z-score for each of the speed and distance parameters was calculated using the formula in Section 2.3. A Z-score higher than 5 and lower than –5 was defined as significantly different from the control cell line (dashed lines). The symbols of the individual parameters are connected with a line. This was not intended to indicate any relationship between the individual parameters, but only serves to improve the interpretability of the data presented. The traveled distance seemed not to be affected. Two cell lines showed a reduced and one cell line an increased speed, whereas the transport of this cell line was less efficient (Confinement Ratio). (**d**) Bar graph of the track duration showed a significant increase in Mut A and C. (**e**) Bar graph of the calculated amount of stops/track. All NPC-deficient cell lines showed a significantly increased number of stops. Graphs in (**b**), (**d**), and (**e**) show mean ± SD with *N* = 4, *n* = 15–17. One-way ANOVA and Dunnet’s multiple comparison post hoc test were used to determine statistical significance, with * = *p* < 0.05, ** = *p* < 0.01, *** = *p* < 0.001. ctrl = control cell line. Mut A (NP-C1): c.1180T>C; Mut B (NP-C1): c.1836A>C/c.1628delC; Mut C (NP-C1): c.3182T>C; Mut D (NP-C2): c.58G>T/c.140G>T.

**Figure 7:**
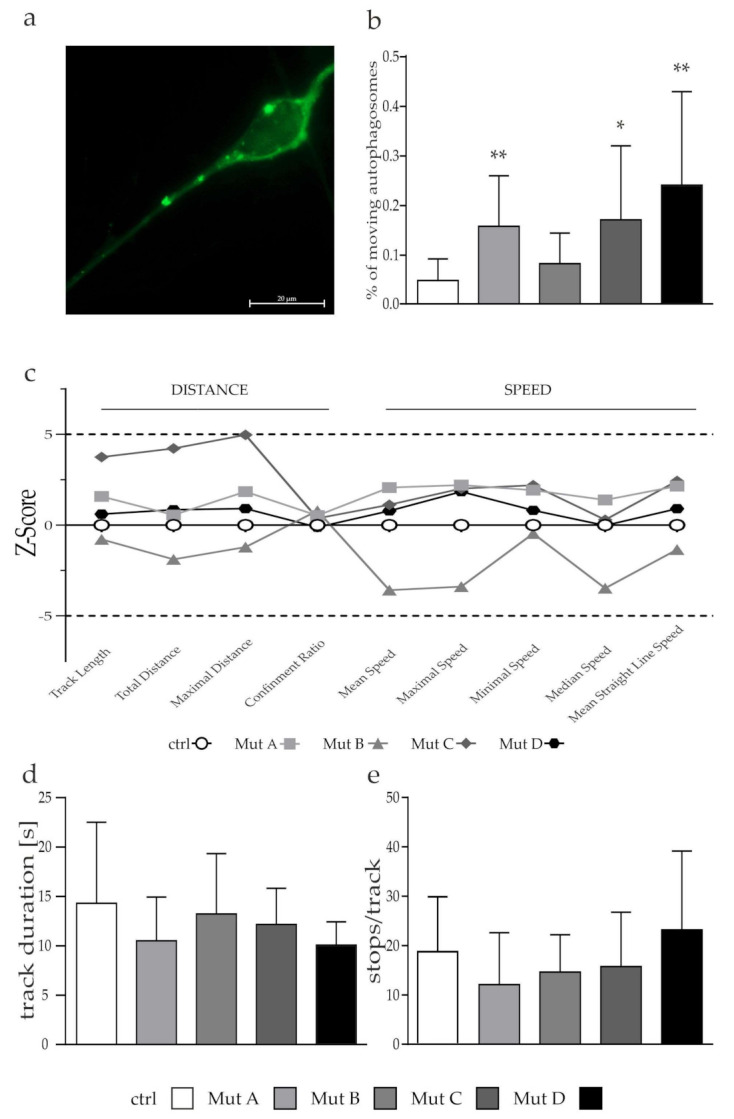
Analysis of autophagosomal transport. (**a**) Example picture of cells transduced with the Premo^TM^ Autophagy Sensor LC3B-GFP. Scalebar = 20 µm (**b**) NPC-deficient cells displayed a significantly increased percentage of moving autophagosomes, with the exception of Mut B. (**c**) Z-score for speed and distance parameters. Dashed lines indicate a Z-score higher than 5 and lower than –5, set as significance levels. The symbols of the individual parameters are connected with a line. This was not intended to indicate any relationship between the individual parameters, but only serves to improve the interpretability of the data presented. (**d**) Bar graph of the track duration and (**e**) stops per track were not different from the control cell line. Graphs in (**b**,**d**,**e**) show mean ± SD with *N* = 4–5, *n* = 17–23. One-way ANOVA and Dunnet’s multiple comparison post hoc test were used to determine statistical significance, with * = *p* < 0.05, ** = *p* < 0.01. Mut A (NP-C1): c.1180T>C; Mut B (NP-C1): c.1836A>C/c.1628delC; Mut C (NP-C1): c.3182T>C; Mut D (NP-C2): c.58G>T/c.140G>T.

A hampered fusion of autophagosomes and lysosomes might be due to an impeded transport of these organelles, since for the fusion process, both need to be in close proximity to each other. As we observed defects in the mitochondrial transport, we assumed similar defects in the transport of autophagosomes and lysosomes affecting their localization, and thus their fusion. Similar to the experiments done for mitochondria, we performed live-cell imaging of cells transduced with the CellLight^TM^ BacMam 2.0 technology for lysosomes (Lamp2-RFP; Figure 6a) and autophagosomes (LC3B-GFP; Figure 7a). The same approach to set a cut-off for stationary and moving organelles was used as described in Section 2.3, leading to thresholds of 0.78 µm for lysosomes and 1.12 µm for autophagosomes. 

The percentage of moving lysosomes was reduced in Mut C, whereas the other cell lines showed no differences compared to the control cell line (Figure 6b). We observed a tendency toward a reduced transport efficiency of lysosomes, comparable to the observation we made for mitochondria. We determined a reduced transport speed for Mut C and Mut D, whereas Mut B showed an increase in the transport speed, but a reduced effectiveness of the overall transport (Figure 6c; Confinement Ratio, Mean Straight Line Speed). Mut A showed no differences with the control cell line regarding speed and distance of the transport. Interestingly, all NPC-deficient cells showed a tendency toward an increased track duration (Figure 6d) accompanied by an increased number of stops (Figure 6e). This may point toward an involvement of the lysosomal transport in the phenotype of NPC-deficient cells. To determine the impact of the transport of autophagosomes, we lastly evaluated their transport, as we did for mitochondria and lysosomes. 

Interestingly, the NPC-deficient cell lines showed an increased amount of moving autophagosomes, whereby Mut B failed to meet significance (Figure 7b). In contrast, the evaluation of the transport of autophagosomes (Figure 7c–e) showed no differences between the NPC-deficient cells and the control cell line, assuming a functional transport of these organelles.

Taken together, we found the mitochondria in NPC-deficient cells to be slightly smaller, whereas their shape and function seemed not to be affected. Nevertheless, their transport speed was reduced, potentially due to an increased amount of stops along their track, which might have had an impact on the recycling of mitochondria via mitophagy. The analysis of mitophagy revealed a hampered capability to incorporate damaged mitochondria in autophagosomes, shown by a reduced colocalization after CCCP treatment. This might have been caused by a failure to induce the parkin expression, and consequently its recruitment to mitochondria to target them for degradation. Additionally, the autophagosome production after induction of stress through mitochondrial damage was reduced, whereas under basal conditions, the amount of LC3BII was increased. 

The analysis of autophagy revealed a reduced capability to form new autophagosomes, as well as a hampered p62 expression. Additionally, the defective fusion of autophagosomes and lysosomes suggested an influence of the observed defective lysosomal transport on this process.

The herein obtained results led us to the hypothesis of a hampered mitophagy/autophagy induction machinery together with a defective clearance of autophagic vesicles due to a reduced organelle fusion.

## 4. Discussion

Mitochondria are the central organelles in cells for the maintenance of a multitude of functions, including proper energy supply, protection against reactive oxygen species, calcium buffering, a functional cell metabolism, and the induction of apoptosis. As they regulate many important pathways, it is not surprising that dysfunctional mitochondria are associated with a number of diseases. Due to their high energy demand and tightly regulated metabolism, neurons seem to be particularly vulnerable to changes in the mitochondrial network and its function. Such an impact on neurodegeneration is already described for diseases such as Alzheimer’s disease (AD), Parkinson’s disease (PD), Huntington’s disease (HD), and amyotrophic lateral sclerosis (ALS) [30]. For NP-C1 alterations in the organization of mitochondria, the respiration machinery, a reduced mitochondrial membrane potential, and a reduced ATP level were described in fibroblasts; all of these were accompanied by a significant enrichment in mitochondrial cholesterol [5]. 

### 4.1. Analysis of Mitochondrial Morphology and Function

The above-mentioned studies all showed an involvement of defective mitochondria in the pathophysiology of different diseases, including NP-C. Morphological alterations often give hints on functional defects such as a reduced MMP, respiration, and/or ATP production, which often lead to the induction of apoptosis caused by damaged mitochondria. In contrast to these studies, we found no hints for functional defects of mitochondria, at least with the assays used here (Figure 2), whereas they seemed to be shorter and generally smaller (Figure 1). For NP-C, functional defects of mitochondria are thought to occur due to an altered cellular cholesterol homeostasis, caused by dysfunction of the NPC1 or NPC2 protein, and leading to an altered cholesterol level in the mitochondrial membrane [31,32]. This might be due to an increased activity of metastatic lymph node protein 64 (MLN64) as a compensating mechanism for the reduced NPC1 activity in lysosomes [33]. Besides, MLN64 the NPC2 protein is thought to function in the cholesterol transport from lysosomes and late endosomes to mitochondria independent of NPC1 [34], whereas we found no major differences between the phenotype of NPC1- and NPC2-deficient cells regarding the parameters observed here. Defects in the mitochondrial membrane potential and ATP production have been described for different other neurodegenerative diseases such as PD [35] and AD [36]. Interestingly, most of the other diseases are associated with an increased level of ROS and elevated antioxidative defense system, which we also could recently demonstrate for NP-C1 [22]. A reduced mitochondrial function is sometimes associated with morphological changes of mitochondria. For example, comparable to our results, mitochondria appeared to be smaller and fragmented in HD accompanied with a reduced function, which was also proven for NP-C [4,5,37]. In contrast, the mitochondrial shape was shown to be unaffected in Charcot–Marie–Tooth disease, whereas the function was reduced [38]. For NP-C1, smaller and more round-shaped mitochondria also have been described [4,39], whereas in our cell model, we found the mitochondria indeed to be smaller, but their shape was not different from the control cell line (excluding an involvement of a hampered fusion and fission to this phenotype), and no functional defects could be observed. 

### 4.2. Impact of Hampered Mitochondrial Transport on Mitophagy in NP-C1- and NP-C2-Deficient Cells

Besides the structural and functional alterations of mitochondria, a functional transport is necessary for their proper function. We found a significantly reduced overall transport speed of mitochondria in all NPC1- and NPC2-deficient cell lines, accompanied by an increased track duration, but found no differences in the distance of transport. This pointed toward a model in which mitochondria require a longer time span to reach their predetermined locations, which could be explained by the increased amount of stops we observed. Besides the microtubules, which function as the “railroad system” for organelles, other cytoskeletal proteins such as vimentin and neurofilaments are thought to have an impact on transport processes as an additional control system. An altered vimentin cytoskeleton was reported to have a negative impact on the transport of mitochondria [40], and similarly, the knockout of the neurofilament light chain (NF-L) led to an increased motility [41]. For NP-C1, we recently reported an altered vimentin assembly [42] leading to a more branched structure, which may have had an impact on the mitochondrial transport. Additionally, the transport proteins kinesin and dynein are known to be affected in different neurodegenerative diseases causing an abnormal axonal transport [25]. A symptomatically comparable disease to NP-C is the autosomal recessive spinocerebellar ataxia 20 (SCAR20), which is also caused by the loss or dysfunction of Purkinje cells in the cerebellum. In this disease, it was found that a deficient sorting nexin 14 (SNX14) protein disrupted microtubule organization and mitochondrial transport [9]. Furthermore, a proper function and expression of Miro1, which is also involved in the induction of mitophagy via the PINK–parkin pathway [11], is crucial for mitochondrial transport, and thus axonal transport can be hampered when function or expression of Miro1 is impeded [6]. As these defects in the axonal transport of mitochondria were confirmed for AD, HD, PD, and ALS [8,43], they might be a general feature in neurodegenerative diseases, and further studies are needed to evaluate the reasons for the observed defects, especially with regard to NP-C.

A proper transport of organelles is furthermore necessary for the recycling of mitochondria, which is in turn crucial to maintain a proper energy supply, especially under stress conditions. The observed transport deficiencies (Figure 3) may therefore hint at a reduced capability of the cells to recycle damaged organelles as a response to organelle damage or general stress. The E3 ubiquitin ligase parkin, together with the mitochondrial serine/threonine-protein kinase PINK1, are necessary for the targeting of mitochondria for degradation [44]. The fact that the NPC1- and NPC2-deficient cells were not able to increase the engulfment of mitochondria into autophagosomes (Figure 4b) and the expression of parkin (Figure 4d) after induction of mitochondrial damage supported the hypothesis of problems in the initial steps of mitophagy. Consequently, it would be interesting to see whether the recruitment of parkin to damaged mitochondria also was affected. As not much is known with regard to NP-C, for PD the involvement of Miro1 and the leucine-rich repeat kinase 2 (LRRK2) in mitochondrial transport and their degradation via mitophagy were reported [45,46]. The effect of Miro1 on mitophagy and energy metabolism in PD was thought to be due to its function in the formation of contact sites between the endoplasmic reticulum and mitochondria [47]. As this might link both pathophysiological features, it might be worthwhile to conduct a more detailed analysis of Miro1 and 2 in NP-C. Another link between NP-C and similar diseases might be the characteristic cholesterol accumulation in lysosomes. In an AD mouse model, it was recently described that high intracellular cholesterol levels affected mitophagy at early stages due to a defective recruitment of the autophagy receptor optineurin (OPTN) [48]. Optineurin furthermore induced the autophagosome formation around damaged mitochondria by interaction with LC3B, but not p62/SQSTM1, which was proven in a cell model for an ALS-associated mutation in optineurin [49]. Instead, Matsumoto et al. suggested a model in which optineurin recruits the TANK-binding kinase 1 (TBK1) to the mitochondria, which phosphorylates p62 to induce the autophagosomal engulfment of mitochondria [50]. The NPC-deficient cells in our study probably reached their maximum of autophagosomes and were therefore unable to increase autophagosome formation after induction of mitochondrial damage (Figure 4e). In this context, evaluation of optineurin and TBK1 may lead to new insights into the potentially defective process of mitophagy induction in NP-C. As the mammalian target of rapamycin complex 1 (mTORC1) is known as a master regulator of general autophagy [51] Davis and colleagues analyzed the effect of mTORC1 with regard to mitochondrial function and mitophagy in NP-C1. They found a hyperactivity of mTORC1 to be the reason for a disrupted mitochondrial function and a decreased proteolytic activity of lysosomes causing a defective mitophagy in a neuronal cell model of NP-C1. Correction of mTORC1 activity, but not the cholesterol accumulation, reduced the observed phenotype, suggesting a role of the cholesterol upstream of mTORC1 [52]. A direct interaction between optineurin and mTOR was recently suggested by Ibrahim and colleagues, as they found a reduced autophagy induction via the mTOR/Unc-51-like kinase (ULK-1) pathway in optineurin knockout mice that was mediated by the group I metabotropic glutamate receptor 5 (mGluR5) [53]. Interestingly, the amount of mGLuR5 receptor in lipid rafts of the cell membrane was described to be reduced in NPC1^−/−^ mice due to the defective cholesterol homeostasis [54], possibly indicating an interaction between cholesterol, optineurin, mTOR, and autophagy/mitophagy. 

### 4.3. Impairement of Autophagy in NPC1- and NPC2-Deficient Cells

The finding of impaired mitophagy in NP-C prompted us to extend our work to an analysis of autophagy in general, as both pathways share many similarities in their initiation and progression. For NP-C, an increased amount of LC3BII associated with an increased amount of autophagosomes was reported due to a defective clearance of autophagic vesicles [55,56]. Besides the increased amount of LC3BII (Figure 5a,b), we observed an altered p62 expression (Figure 5c,d) and a reduced fusion of autophagosomes and lysosomes (Figure 5e,f), displaying a potential reason for the accumulation of LC3BII. The fusion of autophagosomes and lysosomes is a crucial step during the degradation process as autophagosomes themselves are not able to degrade the cargo.

During autophagy induction, the cytosolic LC3BI is converted to LC3BII, which is then incorporated in the autophagosomal membrane. The amount of LC3BII in the autophagosomal membrane might also have an impact on the fusion process with lysosomes [57]. The increased amount of LC3BII might be caused by a nonfunctional recycling of LC3BII from the autophagosomal membrane to the cytosolic LC3BI. It was reported that an altered activity of the autophagy-related protein 4 (ATG4), which is one of the proteins responsible for the conversion of the two LC3B isoforms, resulted in a reduced fusion of the two organelles due to an increased amount of LC3BII [58,59,60]. Interestingly, it was also reported that the function of ATG4 was affected by reactive oxygen species [61]. As these are known pathophysiological features of NP-C and different other neurodegenerative diseases, this might be a possible explanation for the defects in autophagy/mitophagy we observed [22]. Similar to mitochondria, the membrane composition of autophagosomes and lysosomes is important for a functional fusion. It was recently found that a reduction in the cholesterol level in lysosomes, achieved in NPC1-deficient cells treated with the anionic lipid lysobisphosphatidic acid (LBPA), led to a reduction in the LC3BII level, suggesting an improved fusion of the two organelles, and thus an enhanced degradation machinery [62].

Another protein linked to mitophagy/autophagy is p62. Similar to parkin, it targets cargo for degradation via autophagy. We found a less effective p62 expression in NPC-deficient cells when blocking the clearance of autophagic vesicles (AVs) (Figure 5c,d), whereas no differences under basal conditions could be detected, suggesting that the initial targeting of the cargo might have been affected due to a hampered expression of p62. This underlined our hypothesis of a defective autophagy-induction machinery, in addition to defects in the degradation steps. However, this was in contrast to previous findings in NPC1-deficient fibroblasts in which an increased p62 level was reported. In addition, in this study, after a treatment with BafA1, no differences in the p62 level could be detected, pointing to defects in the clearance of AVs [12]. As described above, an involvement of p62 and its phosphorylation status in the targeting of mitochondria during parkin-dependent mitophagy was found recently [50]. This might link our findings of a reduced parkin and p62 expression, as parkin is necessary to recruit p62 to damaged mitochondria. In contrast to our findings, an influence of p62 on autophagosome formation was suggested by Liao and colleagues [63]. They described a decreased amount of LC3BII and autophagosomes in a p62 knockout model after induction of oxidative stress with cisplatin, whereas we found an increased amount of LC3BII. Additionally, the expression of the antioxidative protein nuclear factor erythroid 2-related factor 2 (Nrf2) was reduced under these conditions, which implied a role of p62 in the antioxidative defense system. They also supposed a positive-feedback loop, as the p62 expression was also reduced when Nrf2 was knocked out [63]. In the end, this might link our recent findings of a reduced antioxidative defense system in NP-C with the herein-observed defects in autophagy [22]. Likewise, p62 is thought to influence mitochondrial biogenesis, as the downstream target peroxisome proliferator-activated receptor gamma coactivator 1-alpha (PGC-1α) of Nrf2 has been described as a master regulator of mitochondrial biogenesis [64].

Prior to the fusion of autophagosomes and lysosomes, a proper transport and localization of these organelles is necessary to locate them close to each other. We evaluated the transport kinetics of both organelles, and found the lysosomal transport to be slightly affected, shown by a reduced speed and/or general effectiveness of the transport (Figure 6c–e) in some of the NPC-deficient cell lines. It is therefore reasonable to assume that this might affect the positioning and localization of lysosomes. It was reported that the localization of lysosomes impacted not only the initiation of autophagy, but also the degradation steps. Initially, mTORC1 is recruited to peripheral lysosomes, leading to its activation, and consequently autophagy inhibition, in the step of autophagosome formation. In contrast, a perinuclear localization of lysosomes induced autophagy and facilitated the fusion with autophagosomes [16]. For NP-C1, it was recently shown in an *NPC1^−/−^* mouse model that the increased cholesterol level in lysosomes led to a reduced anterograde transport due to nonfunctional kinesin-1. This was thought to lead to an accumulation of AVs in the distal part of the axons due to an affected autophagosome maturation along their way back to the soma. Interestingly, their transport was not altered, which was consistent with our findings (Figure 7) [14]. As we did not distinguish between retrograde and anterograde transport in our study, a more detailed evaluation of this potential phenotype is necessary to prove the idea that mainly the anterograde transport is affected. In addition, autophagosomes have been found to be capable of retrograde transport by forming so-called amphisomes through fusion with late endosomes, mediated by soluble N-ethylmaleimide sensitive factor attach receptors (SNARE proteins), thus allowing them to use an alternative transport system [65]. Since this depends on functional organelle fusion, which is thought to be impaired in NP-C, it would be of interest to perform a more detailed analysis of the fusion of different organelles during autophagy.

## 5. Conclusions

Taken together, our results supported a model of a defective mitophagy induction machinery in NP-C. We assumed that the reduced mitochondrial transport, as well as a dysfunctional targeting of damaged organelles by parkin and consequently a reduced capability to engulf mitochondria in autophagosomes to initiate their degradation, were the main reasons for that. This might have been due to the altered cholesterol homeostasis affecting the mTORC1 pathway and the recruitment of OPTN, and therefore mitophagy. A hampered LC3B conversion and recycling furthermore contributed to this phenotype by affecting the fusion of autophagosomes and lysosomes, leading to a general accumulation of autophagosomes. This could additionally have been affected by altered transport of lysosomes, leading to potential defects in localization prior to the fusion process. Thus, we concluded that any strategy to improve the initiation and progression of degradative processes such as autophagy/mitophagy might display a successful intervention strategy for NP-C. Additionally, a reduction in the transport phenotype might be beneficial in this processes. Besides, it would be interesting to evaluate the effect of cholesterol reduction treatment on the described kinetic parameters to elucidate the potential link between cholesterol accumulation and organelle function and transport, and to address whether a hampered organelle transport is the reason or the result of the reduced autophagy/mitophagy.

## Data Availability

The data presented in this study are available on request from the corresponding author.

## Supplementary Materials

The following supporting information can be downloaded at: https://www.mdpi.com/article/10.3390/cells11030507/s1, Figure S1: Representative fluorescence pictures of Tom20 stainings; Figure S2: Representative fluorescence pictures of JC-10 stainings; Figure S3: Representative fluorescence pictures of cells transduced with CellLightTM BacMam; Figure S4: Representative fluorescence pictures of cells transduced with the Premo™ Autophagy Tandem Sensor RFP-GFP-LC3B.

## Abbreviations

AD	Alzheimer’s disease
ALS	Amyotrophic lateral sclerosis
ATG4	Autophagy-related protein 4
ATP	Adenosine triphosphate
AVs	Autophagic vesicles
BafA1	Bafilomycin A1
BSA	Bovine serum albumin
CCCP	Carbonyl cyanide 3-chlorophenylhydrazone
DMEM	Dulbecco’s Modified Eagle’s Medium
DMSO	Dimethyl sulfoxide
EGF	Epidermal growth factor
FGF2	Basic fibroblast growth factor
GAPDH	Glyceraldehyde-3-phosphate dehydrogenase
GFP	Green-fluorescent protein
HD	Huntington’s disease
Hsp60	Heat-shock protein 60
iPSC	Induced pluripotent stem cell
Lamp2	Lysosome-associated membrane protein 2
LBPA	Lysobisphosphatidic acid
LC3BI/II	Microtubule-associated proteins 1A/1B light chain 3B
LRRK2	Leucine-rich repeat kinase 2
LSM	Laser scanning microscope
mGluR5	Group I metabotropic glutamate receptor 5
Miro1/2	Mitochondrial rho GTPase 1 or 2
MLN64	Metastatic lymph node 64 protein
MMP	Mitochondrial membrane potential
mTORC1	Mammalian target of rapamycin complex 1
NDCs	Neuronal differentiated cells
NF-L	Neurofilament light chain
NP-C 1/2	Niemann–Pick disease type C 1/2
NPCs	Neural progenitor cells
Nrf2	Nuclear factor erythroid 2-related factor 2
OPTN	Optineurin
PBS	Phosphate-buffered saline
PD	Parkinson’s disease
PFA	Paraformaldehyde
PGC-1α	Peroxisome proliferator-activated receptor-gamma coactivator 1-alpha
PINK1	Serine/threonine kinase PTEN-induced kinase 1
PSA-NCAM	Polysialylated-neural cell adhesion molecule
RFP	Red-fluorescent protein
ROS	Reactive oxygen species
SCAR20	Spinocerebellar ataxia 20
SNARE	Soluble N-ethylmaleimide-sensitive-factor attachment receptors
SQSTM1/p62	Sequestosome-1
SNX14	Sorting nexin 14
TBK1	TANK-binding kinase 1
Tom20	Mitochondrial import receptor subunit TOM20 homolog
ULK-1	mTOR/Unc-51-like kinase
