# Supplementary material for: Impact of Organelle Transport Deficits on Mitophagy and Autophagy in Niemann–Pick Disease Type C
Source: Cells. 2022 Feb 1;11(3):507. doi: 10.3390/cells11030507 (PMC8833886; doi:10.3390/cells11030507)
Supplement: Supplementary file 1 [file cells-11-00507-s001.zip › cells-1576888-supplementary.pdf]

cells

MDPI

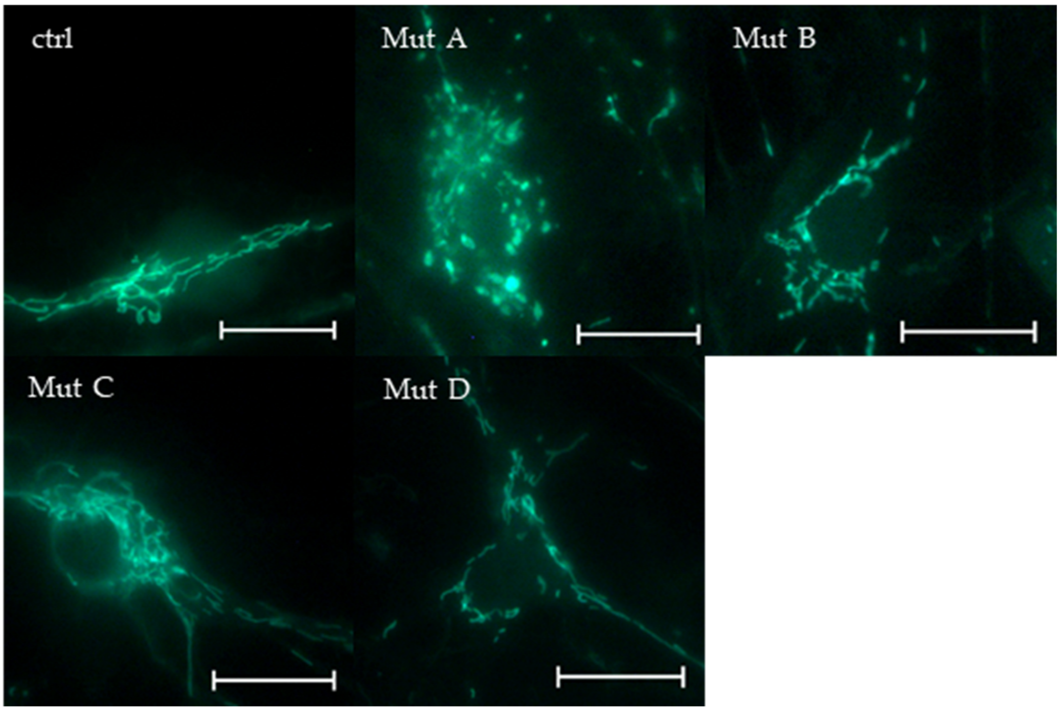

Supplementary Figure S1: Representative fluorescence pictures of cells stained for Tom20 of each cell line used. Mut A (NP-C1): c.1180T>C; Mut B (NP-C1): c.1836A>C/c.1628delC; Mut C (NP-C1): c.3182T>C, Mut D (NP-C2): c.58G>T/c.140G>T. Scalebar 10 µm.

cells MDPI

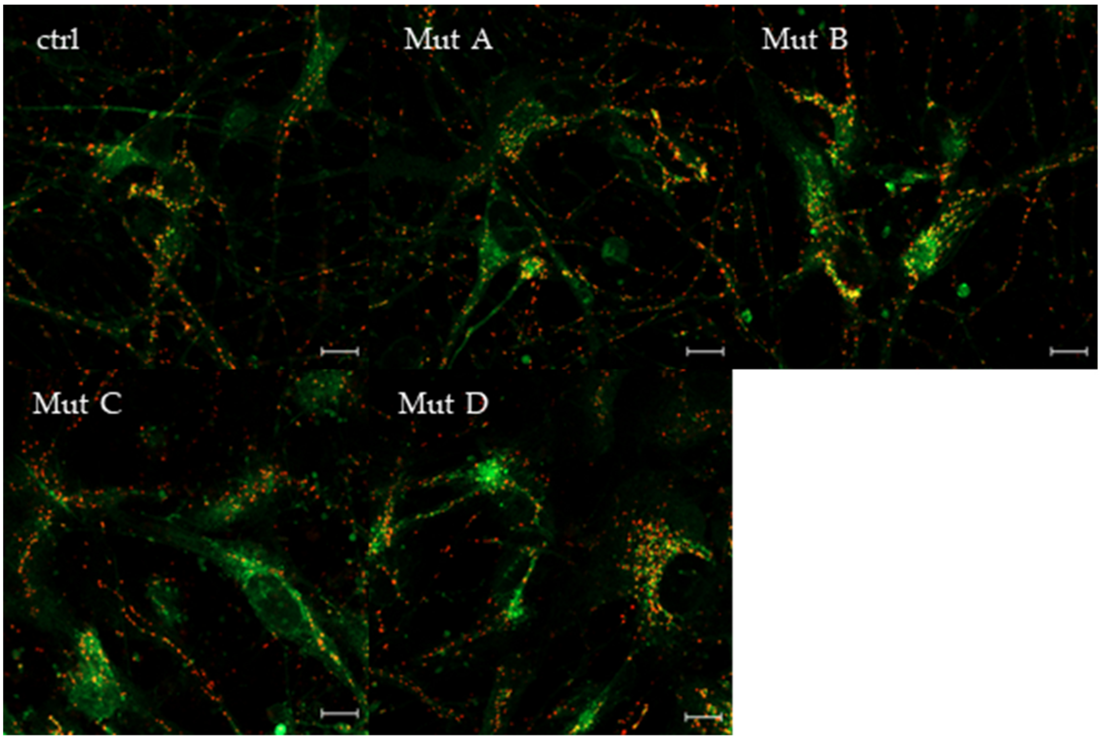

Supplementary Figure S2: Representative fluorescence pictures of cells stained with JC-10 of each cell line used. Mut A (NP-C1): c.1180T>C; Mut B (NP-C1): c.1836A>C/c.1628delC; Mut C (NP-C1): c.3182T>C, Mut D (NP-C2): c.58G>T/c.140G>T. Scalebar 10 µm.

cells MDPI

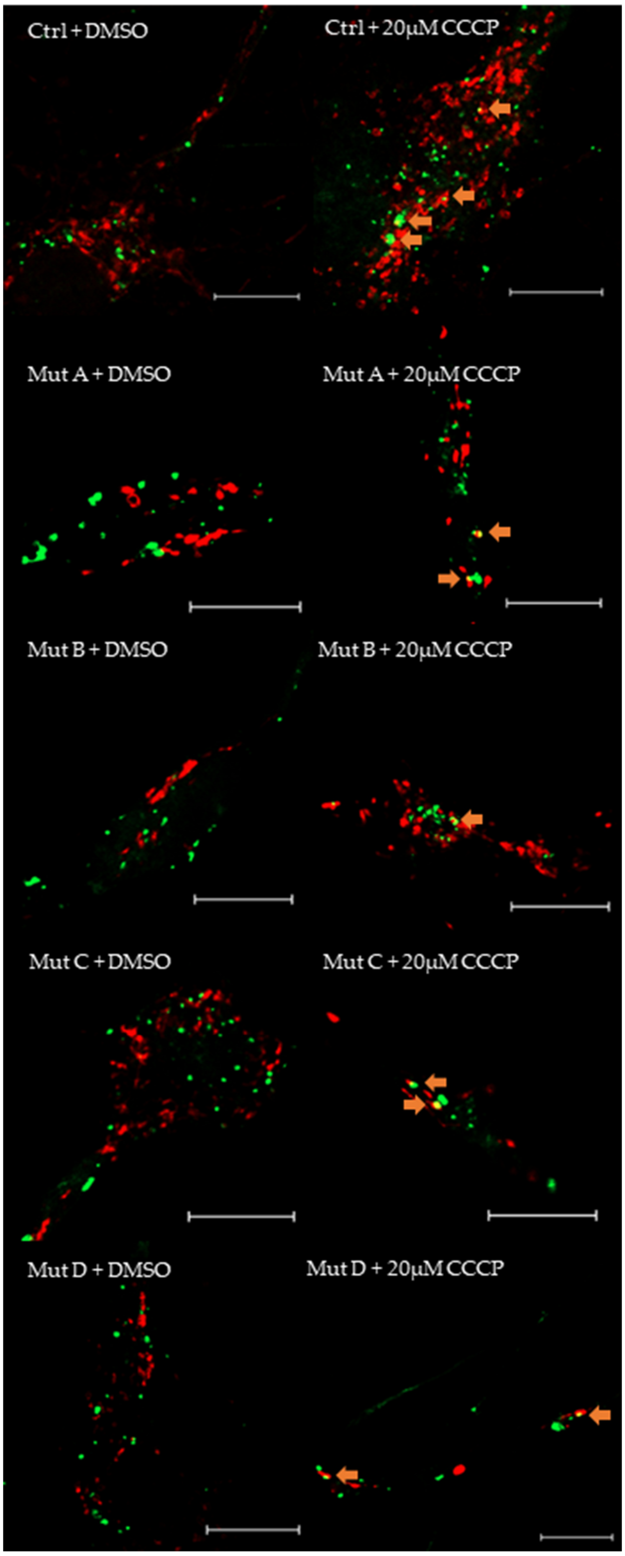

Supplementary Figure S3: Representative fluorescence pictures of cells transduced with CellLight™ BacMam 2.0 technology of each cell line used. Cells were transduced with a Mitochondria-RFP construct to detect mitochondria (red) and with a LC3B-GFP construct to detect autophagosomes (green), to analyze the engulfment of mitochondria by autophagosomes. Pictures show the basal condition (DMSO) and the treatment with CCCP. Mut A (NP-C1): c.1180T>C; Mut B (NP-C1): c.1836A>C/c.1628delC; Mut C (NP-C1): c.3182T>C, Mut D (NP-C2): c.58G>T/c.140G>T. Scalebar 20 µm.

cells MDPI

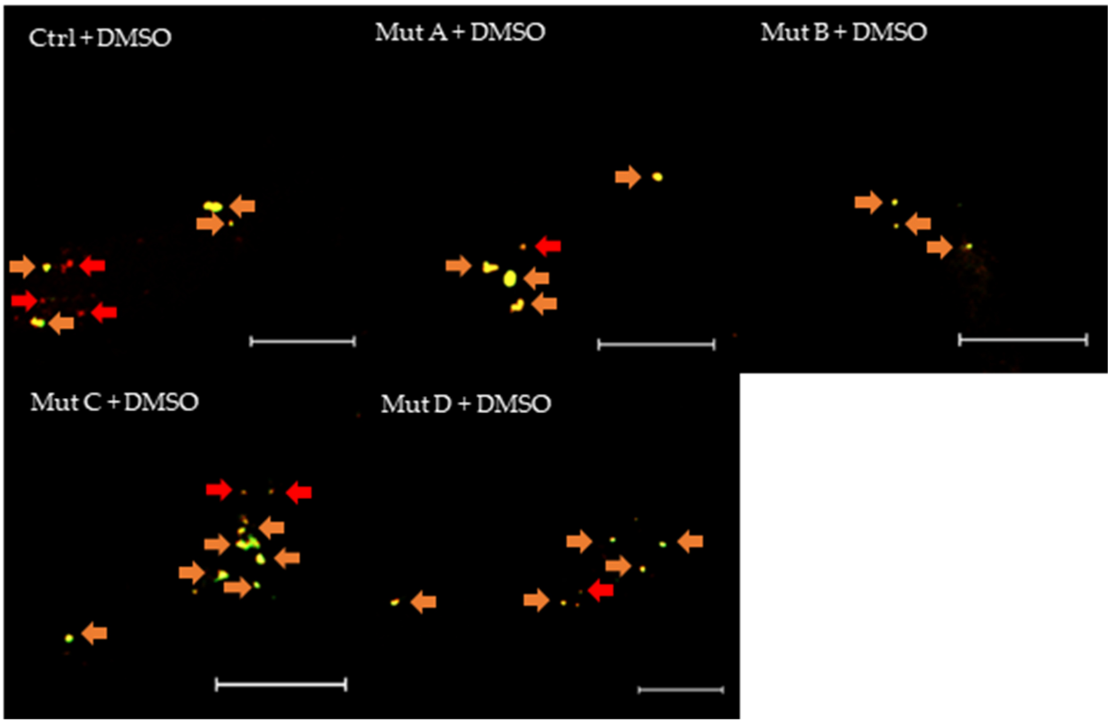

Supplementary Figure S4: Representative fluorescence pictures of cells transduced with the Premo™ Autophagy Tandem Sensor RFP-GFP-LC3B of each cell line used. Pictures were taken under basal conditions (DMSO) and show the formation of autolysosomes (red) and unfused autophagosomes (orange). Mut A (NP-C1): c.1180T>C; Mut B (NP-C1): c.1836A>C/c.1628delC; Mut C (NP-C1): c.3182T>C, Mut D (NP-C2): c.58G>T/c.140G>T. Scalebar 20 µm.
